# Therapeutic electric stimulation does not affect immune status in healthy individuals – a preliminary report

**DOI:** 10.1186/1475-925X-11-42

**Published:** 2012-07-28

**Authors:** Andreja N Kopitar, Vladimir Kotnik, Gaj Vidmar, Alojz Ihan, Primoz Novak, Martin Stefancic

**Affiliations:** 1 Institute of Microbiology and Immunology, Faculty of Medicine, University of Ljubljana, Vrazov trg 2, 1000, Ljubljana, Slovenia; 2 University Rehabilitation Institute, Linhartova 51, 1000, Ljubljana, Slovenia; 3 Institute for Biostatistics and Medical Informatics, Faculty of Medicine, University of Ljubljana, Vrazov trg 2, 1000, Ljubljana, Slovenia

**Keywords:** Electric stimulation, Quadriceps femoris muscle, Immune response, Healthy volunteers

## Abstract

**Background:**

Neuromuscular electric stimulation is widely used for muscle strengthening in clinical practice and for preventative purposes. However, there are few reports on the effects of electric stimulation on the immune response of the organism, and even those mainly describe the changes observed immediately after the electrotherapeutic procedures. The objective of our study was to examine the possible immunological consequences of moderate low-frequency transcutaneous neuromuscular electric stimulation for quadriceps muscle strengthening in healthy individuals.

**Methods:**

The study included 10 healthy volunteers (5 males, 5 females, mean age 37.5 years). At the beginning and after a two-week electric stimulation program, muscle strength was measured and peripheral blood was collected to analyse white blood cells by flow cytometry for the expression of cell surface antigens (CD3, CD19, CD4, CD8, CD4/8, DR/3, NK, Th reg, CD25 + CD3+, CD25 + CD4+, CD25 + CD8+, CD69 + CD3+, CD69 + CD4+, CD69 + CD8+) and phagocytosis/oxidative killing function.

**Results:**

Muscle strength slightly increased after the program on the dominant and the nondominant side. No statistically or clinically significant difference was found in any of the measured blood and immune cells parameters as well as phagocytosis and oxidative burst function of neutrophil granulocytes and monocytes one day after the program.

**Conclusions:**

The program of transcutaneous low-frequency electric stimulation slightly strengthened the quadriceps femoris muscle while producing no changes in measured immunological parameters. Hence, therapeutic low-frequency electric stimulation appears not to be affecting the immune response of healthy persons.

## Background

The use of low-frequency electric stimulation for therapeutic purposes has been increasing during the last decades [1]. In physiatric clinical practice, peripheral electric stimulation is mostly used as neuromuscular electric stimulation (NMES) to improve muscle performance [2,3], but also for pain management [4,5], for healing process enhancement in bone and chronic wounds [6,7], and for increasing blood circulation [8,9]. There are several known contraindications for electric simulation – cardiac arrhythmias, cardiac pacemakers, pregnancy in women, malignant tumours and many other [10,11]. At the same time, numerous physiological effects of electric stimulation have been noted [12,13], as well as psychological ones [14,15].

In recent years, much effort is being invested in clarifying possible health risk of extremely low frequency (ELF) electromagnetic fields from the environment and from different man-made sources. There are increasing concerns of possible effects of different sources of electromagnetic fields on human health, one of them being high-voltage power lines [16]. At the same time, many medical diagnostic and therapeutic applications of ELF fields operate in the same frequency range, but of course with quite different electric field strength [17]. There is also evidence on the connection between certain electrotherapeutic regimes and the nervous-endocrine-immune interactions [18], whereby proper functioning of the immune system is essential for good health.

Our study deals with NMES and its possible effects upon the immune status of the stimulated person. NMES has been used as a complementary tool to therapeutic exercise for muscle strengthening either in clinical practice or for preventative purposes for many years. However, NMES alone yields no higher benefits than traditional strengthening methods [19]. Given the widespread use of NMES, there are surprisingly few reports on the effects of such electric stimulation on the immune response of the organism. The existing reports, which are listed and considered in detail in the discussion, describe either the changes in white blood cells observed immediately after the electrotherapeutic procedures or in a few hours afterwards, or changes in circulating neuroendocrine and inflammatory mediators after some weeks of electric stimulation. We could not find any studies addressing eventual changes in phagocytosis and/or oxidative killing function in the days following the completion of a NMES program on healthy persons, which is why we performed such a study.

## Methods

### Participants

The participants of the study were randomly selected healthy working adults, five women and five men, aged between 25 and 67 years (mean 37.5 years, SD 12.6 years). They had had no electrotherapy for six months prior to inclusion in the study. Upon inclusion in the study, the participants self-assessed the grade of their physical activity (PA) during the last 4 weeks on a 7-point scale (1 = none or little; 2 = light or moderate PA, but not every week; 3 = light PA every week; 4 = moderate PA for less than 30 min every week or light PA 5 times/week; 5 = heavy PA for less than 20 min every week or moderate PA 3 times/week; 6 = moderate PA for at least 30 min at least 5 times/week; 7 = heavy PA for at least 20 min a least 3 times/week). The grade ranged from 3 to 7, with a mean of 5.4. The participants did not change their habits regarding PA during the study – their self-ratings were the same at the end of the study.

All participants signed an informed consent statement. The study was approved by the institutional Research Ethics Committee.

### Neuromuscular electric stimulation

Knee extensor muscles (m. quadriceps femoris) were electrically stimulated on both legs for 10 consecutive working days. The participant selection criteria ensured that all contraindications for electric stimulation [10,11] were respected and the necessary safety measures [20] were undertaken. A two-channel electric stimulator (FEDA PO 32, Soca oprema, Ljubljana, Slovenia) with a voltage outlet was used. The stimulator produces monophasic rectangular pulses of 0.3 ms width and frequency of 20 Hz. The intensity of electric stimulation was set to remain under the pain threshold and ranged from 30 to 50 V (PP) or from 6 to 8 V (RMS), respectively. This was sufficient to elicit light isotonic contraction of the stimulated musculature. The pair of electrodes (self-adhesive rectangular surface electrodes of size 4.5 × 9.0 cm, Valutrode Lite, Neurostimulation Electrodes, Model VL4595, Axelgaard Manufactoring, USA) were fixed to the frontal part of the thigh, with one electrode placed between the upper and the middle third of the thigh and the other electrode placed between the middle and the lower third of the thigh. The electric stimulation was cyclic (7 s of stimulation alternated with 7 s of pause, reciprocally with respect to the dominant/nondominant leg), lasting for 20 min daily. The participants were stimulated in lying position on the back with slightly flexed knees (an angle of about 30°).

Each participant underwent bilateral knee extensor muscle strengthening training using NMES for 10 consecutive working days within a two-week period. On the day before the start of the training program and after the training, knee extensor muscle strength was measured as maximum torque of isometric contraction during voluntary extension of the knees. Before the start of the NMES training (in the morning of the day of the first session) and one day after the end of the training peripheral blood samples were taken for determining biochemical and immune response markers.

### Dynamometric measurements of muscle strength

The measurements of muscle strength (in Nm) were performed on a Biodex System 2 dynamometer. The participants were seated with knees fixed in the flexion position (60°). Maximum voluntary isometric contraction (MVIC) was measured for 5 s with 3 repetitions separated by 20 s breaks. The measurements were performed first on the dominant and then on the nondominant side. The mean MVIC of knee extensor muscles from the 3 repetitions was used for analysis.

### Blood tests

The blood samples were obtained from the cubital vein taken prior to starting of the treatment with electric stimulation (on the same day of the first session in the morning) and after completion of the treatment (in the morning after the last session). The BD Diagnostics Vacutainer Plastic tubes were used to get samples of serum, plasma or whole blood for the laboratory analyses.

The following blood tests were performed:

· Biochemical determination of sodium, potassium, calcium, chlorine, glucose, urea, creatinine, creatine kinase, cortisol and C-reactive protein (CRP);

· Differential white blood cell count (leucocytes, lymphocytes, neutrophils, monocytes);

· Determination of lymphocytes with following characteristics: CD3, CD19, CD4, CD8, CD4/CD8, HLA-DR/CD3, CD56, CD25++CD4+ (Th reg), CD25 + CD3+, CD25 + CD4+, CD25 + CD8+, CD69 + CD3+, CD69 + CD4+, CD69 + CD8+;

· Phagocytosis test of neutrophil granulocytes with FITC labelled E. coli (FTg), phagocytosis test of monocytes with FITC labelled E. coli (FTm);

· Oxidative burst test of E. coli stimulated neutrophil granulocytes with dihydrorhodamine 123 (Ecg);

· Oxidative burst test of E. coli stimulated monocytes with dihydrorhodamine 123 (Ecm).

### Acquisition of immunologic data

Determination of potential activation changes in three white blood cell groups- lymphocytes, neutrophil granulocytes and monocytes - was performed. Lymphocytes were analysed for the expression of cell surface antigens using the following combination of antibodies HLA-DR-FIRC/CD3-PE, CD25-FITC/CD3-PE/CD8-PerCPCy5.5/CD4-APC, CD69-FITC/CD3-PE/CD8-PerCP Cy5.5/CD4-APC, all from BD Biosciences (Oxford, UK) and CD3-PE, CD4-PE Cy5, CD8-FITC, CD19-FITC, CD56-PE Cytognos (Salamanca, Spain), and neutrophil granulocytes and monocytes for phagocytic function (FTg, FTm) and oxidative burst ability (Ecg and Ecm).

### Blood count

Standard blood count was performed to determine the number of white cells per L of blood in the sample. Differential count was done to acquire information on the per-cent-distribution of specific white blood cells. Both data were used to calculate the absolute number of a specific white cells population for each single individual.

### Flow cytometry

White blood cells were stained with specific monoclonal antibodies directed against different CD markers. Four colour analyses were performed concurrently to determine the population of cells under investigation and their function. At least 1000 gated cells were analysed for each test. Data were collected on a FACSCanto flow cytometer and expression of various markers was assessed using FACSDiva (BD Bioscience) analysis software.

### Phago-Burst Test

Phagocytosis and oxidative burst capacity was measured using Phago-burst test (Orphegen Pharma, Heidelberg, Germany). Measurements were performed using flow cytometer (BD, FACSCanto, USA) equipped with argon-ion laser of 488 nm excitation wavelength.

### Statistical analysis

Descriptive statistics were calculated for all the measured parameters. Because of the small sample size and the exploratory nature of the study, the change in MVIC between the start and the end of the NMES program within each side, the difference between sides in mean change, and the change between pre- and post-treatment data from blood tests were tested both using paired *t*-test and exact Wilcoxon signed-rank matched-pairs test (WSRMPT). For biochemical blood tests, the difference in proportion of values within normal range was tested using exact McNemar's test. Because of the exploratory nature of the study, no adjustment for multiple testing was performed, so all the differences were taken as statistically significant if *p* < 0.05. Statistical analyses were performed using SPSS for Windows 15.0.1.1 software (SPSS Inc.. Chicago. IL. 2007).

## Results

### Dynamometric measurements of muscle strength

Measurements of knee extensor muscles strength before and after the NMES program are summarised in Table 1. The increase in mean MVIC was statistically significant on the dominant as well as on the nondominant side (*p* < 0.01 on both sides from *t*-test and WSRMPT), though not very large (by 6 % and 5 % on average on the dominant and the nondominant side, respectively). There was no difference between the sides in mean MVIC increase (*p* = 0.979 and *p* = 1.000 from *t*-test and WSRMPT, respectively).

**Table 1:** Descriptive statistics for maximum voluntary isometric contraction (MVIC) measured before and after the 2-week neuromuscular electric stimulation program (NMES), and for the difference and the ratio of the two measurements

	**Dominant side**				**Nondominant side**			
**MVIC (Nm)**	**Before NMES**	**After NMES**	**Difference**	**Ratio**	**Before NMES**	**After NMES**	**Difference**	**Ratio**
Min	117	133	−2	0.99	134	142	−4	0.97
Max	328	344	30	1.21	339	353	22	1.11
Median	219.5	225.5	11.5	1.04	210.0	230.5	15.0	1.06
Mean	220.4	232.4	12.0	1.06	218.6	230.5	11.9	1.05
SD	80.0	82.2	11.2	0.07	74.7	78.71	8.5	0.04

Difference - Measurement after NMES – Measurement before NMES.

Ratio - Measurement after NMES / Measurement before NMES.

### Biochemical blood tests

The results regarding biochemical serum analyses and electrolytes are summarised in Tables 2 and 3, respectively. Some increase after the NMES program was observed only in creatine kinase, but it was not statistically significant. CRP values were all below the threshold of 5 mg/L, before and after the NMES program in all the participants; therefore, no descriptive statistics are reported for them. Similarly, no systematic change after the NMES program was observed in any of the electrolytes – neither in the mean value nor or in the proportion of normal values.

**Table 2:** Descriptive statistics and results of statistical tests for biochemical serum analyses

	**Glucose**		**Creatine kinase**		**Urea**		**Creatinine**		**Cortisol**	
	**(mmol/L)**		**(μkat/L)**		**(mmol/L)**		**(μmol/L)**		**(mmol/L)**	
	** *Before* **	** *After* **	** *Before* **	** *After* **	** *Before* **	** *After* **	** *Before* **	** *After* **	** *Before* **	** *After* **
	** *NMES* **	** *NMES* **	** *NMES* **	** *NMES* **	** *NMES* **	** *NMES* **	** *NMES* **	** *NMES* **	** *NMES* **	** *NMES* **
**Min**	4.0	4.0	1.10	1.31	3.0	3.9	49.0	55.0	235	324
**Max**	5.6	5.9	7.50	12.81	6.9	7.7	107.0	103.0	889	1044
**Median**	5.0	4.8	2.41	4.00	5.2	5.1	72.0	75.5	450	442
**Mean**	4.9	4.9	3.10	4.83	5.1	5.2	76.3	78.8	479	510
**SD**	0.6	0.5	2.32	3.75	1.3	1.2	18.6	17.0	187	236
** *p* (paired *t* )**	0.788		0.146		0.654		0.185		0.576	
** *p* (EWMPT)**	0.715		0.193		0.531		0.217		0.945	
**Normal**	10 / 10	10 / 10	5 / 10	4 / 10	10 / 10	9 / 10	9 / 10	10 / 10	9 / 10	9 / 10
** *p* (McNemar)**	1.000		0.965		1.000		1.000		1.000	

NMES - neuromuscular electric stimulation program.

Normal - Number of values within normal range / Number of data values.

EWMPT - exact Wilcoxon signed-rank matched-pairs test.

McNemar - exact McNemar test.

**Table 3:** Descriptive statistics and results of statistical tests for electrolytes

	**Glucose**		**Creatine kinase**		**Urea**		**Creatinine**		**Cortisol**	
	**(mmol/L)**		**(μkat/L)**		**(mmol/L)**		**(μmol/L)**		**(mmol/L)**	
	** *Before* **	** *After* **	** *Before* **	** *After* **	** *Before* **	** *After* **	** *Before* **	** *After* **	** *Before* **	** *After* **
	** *NMES* **	** *NMES* **	** *NMES* **	** *NMES* **	** *NMES* **	** *NMES* **	** *NMES* **	** *NMES* **	** *NMES* **	** *NMES* **
**Min**	4.0	4.0	1.10	1.31	3.0	3.9	49.0	55.0	235	324
**Max**	5.6	5.9	7.50	12.81	6.9	7.7	107.0	103.0	889	1044
**Median**	5.0	4.8	2.41	4.00	5.2	5.1	72.0	75.5	450	442
**Mean**	4.9	4.9	3.10	4.83	5.1	5.2	76.3	78.8	479	510
**SD**	0.6	0.5	2.32	3.75	1.3	1.2	18.6	17.0	187	236
** *p* (paired *t* )**	0.788		0.146		0.654		0.185		0.576	
** *p* (EWMPT)**	0.715		0.193		0.531		0.217		0.945	
**Normal**	10 / 10	10 / 10	5 / 10	4 / 10	10 / 10	9 / 10	9 / 10	10 / 10	9 / 10	9 / 10
** *p* (McNemar)**	1.000		0.965		1.000		1.000		1.000	

NMES - neuromuscular electric stimulation program.

Normal - Number of values within normal range / Number of data values.

EWMPT - exact Wilcoxon signed-rank matched-pairs test.

McNemar - exact McNemar test.

### Immunological status

No statistically significant difference between the status before the NMES program and the status after NMES program was found in any of the observed variables (Table 4). The only changes close to statistical significance were the raises in percentage of CD3 cells and number and percentage of CD19 cells.

**Table 4:** Summary of results of blood tests before and after the 2-week neuromuscular electric stimulation program

	** *Before NMES* **		** *After NMES* **		**Before vs. After (no. of cases)**			** *p* **	** *p* **
	**Range**	**Mean**	**Range**	**Mean**	**<**	**=**	**>**	**(paired *t* )**	**(EWMPT)**
**Leucocytes**	3.8 - 8.0	5.7	4.7 - 7.9	5.7	4	1	5	0.901	0.719
**Neutrophils (%)**	36.4 - 67.7	51.8	37.6 - 68.9	52.4	5	0	5	0.888	1.000
**Neutrophils (no.)**	1.4 - 5.4	3.0	2.2 - 5.4	3.1	5	0	5	0.967	0.695
**Monocytes (%)**	2.8 - 7.4	4.1	3.2 - 7.8	4.2	5	1	4	0.943	0.910
**Monocytes (no.)**	0.1 - 0.3	0.2	0.2 - 0.5	0.2	5	0	5	0.894	0.770
**Lymphocytes (%)**	28.3 - 54.6	38.8	25.8 - 51.1	37.9	4	0	6	0.792	1.000
**Lymphocytes (no.)**	1.7 - 2.9	2.2	1.8 - 3.0	2.2	2	0	8	0.725	0.375
**CD3 (%)**	60.0 - 84.0	72.4	61.0 - 87.0	75.6	6	3	1	0.088	0.078
**CD3 (10**^**9**^**cells/L)**	1.012 - 2.423	1.639	1.171 - 2.460	1.686	4	0	6	0.734	0.922
**CD19 (%)**	2.0 - 24.0	8.5	5.0 - 23.0	10.0	7	0	3	0.051	0.059
**CD19 (10**^**9**^**cells/L)**	0.042 - 0.398	0.184	0.102 - 0.442	0.218	8	0	2	0.065	0.064
**CD4 (%)**	31.0 - 56.0	43.5	36.0 - 62.0	47.6	6	2	2	0.128	0.117
**CD4 (10**^**9**^**cells/L)**	0.691 - 1.483	0.997	0.766 - 1.719	1.061	5	0	5	0.473	0.770
**CD8 (%)**	17.0 - 45.0	27.2	15.0 - 50.0	27.1	3	2	5	0.940	0.992
**CD8 (10**^**9**^**cells/L)**	0.282 - 1.298	0.626	0.288 - 1.371	0.616	5	0	5	0.779	0.846
**CD4/8**	0.7 - 2.6	1.7	0.7 - 3.9	1.9	6	1	3	0.289	0.359
**DR/3 (%)**	4.0 - 25.0	9.0	3.0 - 20.0	8.5	4	0	6	0.485	0.561
**DR/3 (10**^**9**^**cells/L)**	0.083 - 0.558	0.207	0.061 - 0.420	0.185	4	0	6	0.302	0.432
**NK (%)**	6.0 - 37.0	13.7	6.0 - 26.0	13.1	4	1	5	0.829	0.633
**NK (10**^**9**^**cells/L)**	0.159 - 0.776	0.303	0.178 - 0.572	0.288	4	0	6	0.780	0.557
**Th reg (%)**	0.8 - 4.0	1.8	0.1 - 5.0	2.1	5	2	3	0.328	0.344
**Th reg (10**^**9**^**cells/L)**	0.014 - 0.051	0.028	0.002 - 0.077	0.036	6	0	4	0.211	0.232
**CD25 + CD3+ (%)**	2.0 - 14.0	6.1	1.0 - 16.0	5.6	4	3	3	0.674	1.000
**CD25 + CD3+ (10**^**9**^**cells/L)**	0.042 - 0.294	0.136	0.018 - 0.325	0.125	5	0	5	0.691	1.000
**CD25 + CD4+ (%)**	3.0 - 24.0	11.5	1.0 - 24.0	11.0	5	2	3	0.805	0.938
**CD25 + CD4+ (10**^**9**^**cells/L)**	0.066 - 0.312	0.183	0.025 - 0.381	0.178	6	0	4	0.964	0.846
**CD25 + CD8+ (%)**	0.1 - 2.0	0.9	0.3 - 2.0	0.8	1	8	1	0.555	1.000
**CD25 + CD8+ (10**^**9**^**cells/L)**	0.001 - 0.034	0.015	0.004 - 0.049	0.016	4	0	6	0.751	1.000
**CD69 + CD3+ (%)**	1.0 - 8.0	2.8	1.0 - 4.0	2.2	3	5	2	0.363	0.625
**CD69 + CD3+ (10**^**9**^**cells/L)**	0.021 - 0.231	0.069	0.018 - 0.088	0.049	4	0	6	0.324	0.922
**CD69 + CD4+ (%)**	1.0 - 4.0	1.7	1.0 - 4.0	2.1	4	5	1	0.168	0.313
**CD69 + CD4+ (10**^**9**^**cells/L)**	0.010 - 0.081	0.031	0.012 - 0.062	0.036	6	0	4	0.412	0.432
**CD69 + CD8+ (%)**	1.0 - 4.0	2.4	1.0 - 4.0	2.0	3	2	5	0.309	0.344
**CD69 + CD8+ (10**^**9**^**cells/L)**	0.010 - 0.097	0.042	0.016 - 0.094	0.035	4	0	6	0.308	0.375
**FTg (%)**	84.3 - 97.1	90.5	84.1 - 97.0	91.5	5	0	5	0.334	0.322
**FTm (%)**	68.7 - 92.1	82.0	80.6 - 93.4	84.3	6	0	4	0.538	0.492
**Ecg (%)**	89.8 - 98.7	93.6	84.0 - 98.9	91.8	5	0	5	0.422	0.375
**Ecm (%)**	77.7 - 95.8	86.0	71.4 - 95.8	87.0	7	0	3	0.632	0.322

EWMPT - exact Wilcoxon signed-rank matched-pairs test.

## Discussion

The main aim of our study was to examine the immune response after the completion of the NMES training. The question at issue was whether the particular type of low frequency therapeutic electric stimulation in healthy individuals, if frequent and long-lasting, has any effect on their immune response. Our results show no statistically or clinically significant effect.

Some studies of this type have been carried out as *in vitro* experiments. A study on immunomodulatory effects of direct and pulsed electric currents found that directly applied weak electric currents can modulate the function of different immune cells *in vitro*[21,22]. In an animal experiment, it has been shown that electric stimulation leads to acceleration of fracture healing attended by corresponding shifts of the relationship between immune processes (levels of IgM, IgG, IgA, T- and B-lymphocytes) and activity of the regenerative processes [23].

There are also some clinical studies of the influence of low-frequency electric stimulation evoking muscle contractions on neuroendocrine changes and on immune reactions in humans. For example, Twist and co-workers examined the effects of computerized functional electric stimulation (FES) exercise program on plasma β-endorphin-like immunoreactivity (BEP-ir) and cortisol levels in spinal cord-injured individuals, and found significantly sustained increases in BEP-ir and improved regulation of cortisol together with improved depression scores after 19 and 30 weeks of training program [24]. Furthermore, Nash reported that cycling exercise performed by persons with quadriplegia using computer-sequenced electrically stimulated contraction of lower leg muscles fails to provoke a leucocytosis, but transitionally elevates natural killer (NK) cell number and citotoxicity lasting one-half hour after exercise [25]. Also, Klokker *et al.* found changes in NK and other immunocompetent cells after 30 minutes of electrically stimulated cycling exercise in spinal cord injured individuals, which mostly returned to pre-exercise level after two hours [26]. In a study in patients with moderate to severe heart failure, Karavidas and co-workers evaluated the impact of FES on endothelial and peripheral markers of immune activation and observed that 6 weeks of FES training program improved endothelial function and exhibited anti-inflammatory effects [27]. In addition, numerous reports deal with the effect of active exercise and physical training upon the immune system [28,29].

In rehabilitation of musculoskeletal system, patients with weakened muscles are frequently encountered, so muscle strengthening is often used. Increase in muscle strength is usually obtained through exercise. In addition to adequately selected and conducted active exercise, another option for muscle strengthening is neuromuscular electric stimulation (NMES). The effects of NMES for the purpose of muscle strengthening have been described in healthy subjects and in several pathologies associated with decrease of muscle strength [2,19]. The results obtained in those studies vary considerably and it is difficult to compare them because of the differences in the applied electro-stimulation procedure, as well as in the measurement of effects upon muscle strength. Many authors describe a raise in muscle strength after NMES training, though only a few detected statistically significant changes with respect to the baseline. The variation among the outcomes is not surprising because the effects of NMES depend on a number of factors, while the studies vary regarding the experimental protocol, equipment used, characteristics of the electric pulses, duty cycles and other relevant aspects [30].

Nevertheless, muscle strengthening using NMES has long been used in clinical practice – mainly in traumatological and orthopaedic patients without lesions of the nervous system, who have weakened yet normally innervated muscles [31,32]. There are also some lesions of the central nervous system leading to sarcopenia which could be treated by NMES, but that needs further evaluation.

NMES is considered as a supplementary method of muscular strengthening added to therapeutic exercise. In addition, NMES superimposed on voluntary muscle contraction can be used in healthy persons for the preventative purposes of maintaining muscular fitness. Given the lack of studies addressing eventual changes in immunological condition in the days following the completion of a NMES program, it is therefore important that our results indicate that therapeutic electric stimulation does not harm immune response in healthy volunteers one day after completion of a 10-session-day NMES program.

It should be stressed that we used the standard contemporary methods for comprehensively assessing immune status. While even more sensitive methods might exist (and are expected to be developed in the future), we can conclude with sufficient certainty that the treatment did not produce any physiologically or clinically significant change in the functioning of the immune system.

From the methodological point of view, the issue of sample size (and study power) should be addressed. Namely, one may be tempted to conclude that the absence of any statistically significant effect was due to the small sample size, but because no correction for multiple testing was applied the *p*-values underestimate the true probability of falsely rejecting the null hypothesis. It becomes clear that such "compensation" is sufficient if one considers that had the Bonferroni adjustment been applied (which is, of course, overly conservative, especially with correlated outcomes like in our study, but suffices for illustrative purposes), all *p*-values in Table 4 would equal 1. It should also be stressed that no formal sample size calculation could have been performed because of the lack of directly comparable previous research, while calculating "post-hoc" ("observed", "retrospective", "achieved") statistical power would not be the right way to address the "sensitivity" of our study [33,34]. In addition to statistical arguments, clinical significance should also be considered. As far as the biochemical serum analyses and electrolytes are concerned, the fact that the proportion of values within the normal range remained practically identical after the NMES program leaves little doubt. Similarly, the three possibly increased immunological status parameters were within the reference range (with negligible exceptions) before and after the program in all the participants, the reference range being 54.9-84.0 % for the percentage of CD3 cells, 4.7-19.1 % for the percentage of CD19 cells, and 0.072-0.460 × 10^9^ cells/L for the number of CD19 cells [35].

Nevertheless, while drawing the conclusion that neuromuscular electric stimulation does not affect immunological status, one should bear in mind that the performed electric stimulation was relatively moderate in terms of intensity of the electric pulses. It is therefore not known what the immune response would be using higher amplitude and/or different frequencies of electric stimulation, treatment of different duration and/or of different tissues.

## Conclusions

Measurements of maximum torque of isometric contraction during maximum voluntary isometric contraction showed that muscle strength slightly increased after ten sessions of a neuromuscular electric stimulation training program. At the same time, we found no statistically or clinically significant change of the biochemical blood parameters or of the immunological status parameters monitored in our study group of healthy persons one day after the treatment with neuromuscular electric stimulation.

## Competing interests

The authors declare that they have no competing interests.

## Authors' contributions

ANK planned and carried out all the biochemical and immunological tests. VK participated in study design and coordination, and helped to draft the manuscript. GV participated in the design of the study, performed the statistical analyses and drafted the manuscript. AI supervised the biochemical and immunological tests, and reviewed the manuscript. PN participated in study design and coordination, and reviewed the manuscript. MS is the senior author who conceived of the study, participated in its design and coordination, and drafted the manuscript. All authors read and approved the final manuscript.
